# Regulation of Calcium Ion Channels with Conjugated Molecules for Modulating Biological Functions

**DOI:** 10.1021/acscentsci.5c01522

**Published:** 2025-10-01

**Authors:** Hao Zhao, Fengting Lv, Shu Wang

**Affiliations:** † Beijing National Laboratory for Molecular Sciences, Key Laboratory of Organic Solids, Institute of Chemistry, Chinese Academy of Sciences, Beijing 100190, P. R. China; ‡ Interdisciplinary Research Center for Chemical, Life and Health Sciences, Institute of Chemistry, Chinese Academy of Sciences, Beijing 100190, P. R. China

## Abstract

Calcium ion (Ca^2+^) channels play a key role in mediating cellular gene transcription and signal transduction, emerging as intriguing targets for modulating biological functions. Conjugated molecules (CMs) exhibit distinctive advantages of tunable optoelectronic properties, intrinsic bioactivities, and flexible assembly characteristics, providing various opportunities for the regulation of Ca^2+^ channels. In this Outlook, we introduce the CM-based external-light-reliable or -free photodynamic, the first/second near-infrared (NIR-I/II) light-enabled photothermal, and supramolecular regulations of Ca^2+^ channels for modulating biological functions, including cancer and thrombolysis therapy, remote neurostimulation, and glycemic management. On the other hand, the challenges and perspectives on advancing fast response time, multimodal responsiveness, targeted precision, and biosafety are also discussed, clearly mapping out future development trajectories.

## Graphical abstract

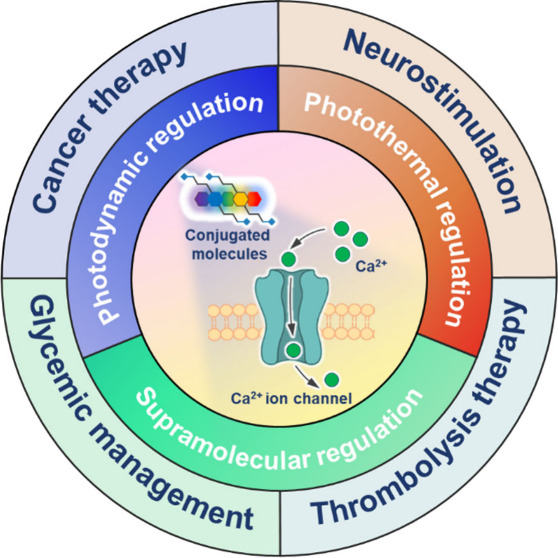

## Introduction

The calcium ion (Ca^2+^) channel is a class of transmembrane proteins that can selectively modulate Ca^2+^ flow across cell membranes.^1^ In particular, Ca^2+^ channels have demonstrated pivotal roles in intra- and/or intercellular activities ranging from gene transcription to signal transduction, thus emerging as attractive targets for precision therapies.^2^ The key for regulation of Ca^2+^ channels is the development of advanced agents and/or methodologies that are able to activate or inhibit Ca^2+^ influx remotely and selectively. In contrast to chemogenetics,^3,4^ gene editing,^5^ electrogenetics,^6^ and mechanogenetics,^7,8^ optogenetics, in which light (including light-induced oxidative stress and/or heat) triggers the activation of genetically engineered proteins, has revolutionized such fields with desirable noninvasiveness, unprecedented resolution, and switchable reversibility, thus allowing for the precise regulation of Ca^2+^ channels in living cells.^9–11^ Nanocrystals of metals (i.e., Au and Cu),^12,13^ upconversion nanoparticles (UCNPs),^14,15^ and carbon nanomaterials^16,17^ have been reported for regulation of Ca^2+^ channels. Nevertheless, they exhibit undefined chemical structures, potential toxicity, and unclear photophysical mechanisms, prohibiting unambitious elucidation of the structure–property relationships and further academic translations. Rational design and development of optical and/or intrinsic active agents are anticipated to enrich the toolbox for regulation of Ca^2+^ channels but have still remained challenging.

Conjugated molecules (CMs), namely, conjugated polymers and/or oligomers herein, are characterized by delocalized π-conjugated backbones.^18,19^ Compared to the inorganic nanomaterials, synthetic CMs can exhibit atomically precise structures, modulable energy levels, biocompatibility as well as hierarchical light harvesting, energy transfer, photosensitization and photoconversion capabilities,^20,21^ providing various opportunities for the interdisciplinary optoelectronic and biological applications.^22,23^ For example, CMs with hydrophilic side chains, corresponding to water-soluble CMs, can optically sensitize surrounding O_2_ to generate reactive oxygen species (ROSs) followed by photodynamically eliminating infectious bacterial and malignant tumors,^24,25^ and a growing number of photothermal CMs nanoparticles have also been obtained to induce high temperature for disease diagnosis and theranostics.^26,27^ Notably, some CMs that are capable of interacting with macrocyclic host molecules have been reported for switchable regulation of their intrinsic membrane-embedding and optical activities,^28,29^ and significant efforts have been recently devoted to realizing biohybrid photosynthesis using CMs as light-harvesting antenna and photogenerated electron pool.^22,30,31^ In short, the intriguing photodynamic, photothermal, and supramolecular bioactivities of CMs are solidly demonstrated, highlighting their prospects for regulation of Ca^2+^ channels, and some milestone research has been consecutively achieved.^32–34^ Despite these successes, there are still significant hurdles toward more practical applications, clearly mapping future development trajectories.

In this Outlook, we aim to provide a concise overview of contemporary research on the regulation of Ca^2+^ channels with CMs for modulating biological functions, including cancer and thrombolysis therapy, remote neurostimulation, and glycemic management. CM-mediated photodynamic effects, either in an external-light-reliable or light-free manner, for regulating Ca^2+^ channels are summarized. We then introduce photothermal manipulation of Ca^2+^ channels using CMs as conversion elements and discuss how this promising strategy can resolve previously unaddressed challenges. Notably, we further emphasize a CM-based supramolecular system that enables switchable regulation of Ca^2+^ channels through controllable modulation of the intrinsic surface potential of CMs without extra stimuli, highlighting the proof-of-concept of “supramolecular genetics”. Especially, we present an outlook with potential development directions of CM-based regulation of Ca^2+^ channels, outlining key development directions that could unlock transformative applications in both fundamental research and clinical therapeutics. It should be noted that this Outlook focuses more on the CMs for regulation of Ca^2+^ channels, summarizing the state-of-the-art, challenges, and perspectives in such fields. We refer the readers to other reviews and/or representative articles for more comprehensive understanding of the existing materials and techniques for regulating ion channels.^10,35–40^

## PHOTODYNAMIC REGULATION OF the Ca^2+^ CHANNEL

ROS-sensitive Ca^2+^ channels, such as transient receptor potential-melastatin 2 (TRPM2), -vanilloid 1 (TRPV1), and -ankyrin 1 (TRPA1), have been revealed to allow for the oxidative-stress-enabled activation, followed by mediating Ca^2+^ influx and the subsequent cascade signal transductions for modulating biological functions.^41,42^ Compared to the direct addition of oxidants (i.e., hydrogen peroxide (H_2_O_2_) and nanocatalyst) to living systems, the optically induced *in situ* generation strategy provides the noninvasiveness, biocompatibility, and high spatiotemporal resolution, holding great promise toward more precise and remote modulations. For example, Xing et al. reported the remote activation of ROS-sensitive TRPM2 channels by near-infrared (NIR)-enabled CM nanoparticles for enhanced cancer therapy (Figure 1a).^43^ Specifically, ETTC, a π-conjugated oligomer with the typical acceptor–donor–acceptor (A–D–A) structure was synthesized, and water-dispersible nanoparticles containing NIR-absorbing ETTC were fabricated with the ROS-cleavable diselenide amphiphilic polymer (DSPE-PEG-SeSe-COOH), which were further surface-functionalized with cationic polyethylenimine (PEI) for electrostatically interacting and thus delivering TRPM2 plasmid. With an intrinsic small singlet–triplet splitting (Δ*E*_ST_) of 0.65 eV, ETTC demonstrated favorable intersystem crossing (ISC). Triggered by 808 nm laser illumination, robust ROSs were generated through the photodynamic effect of ETTC nanoparticles, which further cleaved the Se–Se linkages, releasing the TRPM2 plasmid for improving TRPM2 expression in cancer cells. Simultaneously, ROSs also activated TRPM2 to induce Ca^2+^ influx, which inhibited early autophagy and up-regulated intracellular ROS production, ultimately leading to the death of TRPM2-overexpressing cancer cells both *in vitro* and *in vivo*. Recently, they developed CM nanoparticles with the capability of NIR-triggered *in situ* release of nitric oxide (NO), realizing the activation of TRPV1 for eliminating glioblastoma.^44^ These works provide CM-based photodynamic systems for activating TRPM2 and TRPV1, paving the way to establish new platforms using ROS-sensitive Ca^2+^ channels as promising targets and/or tools for cancer therapy.

**Figure 1:**
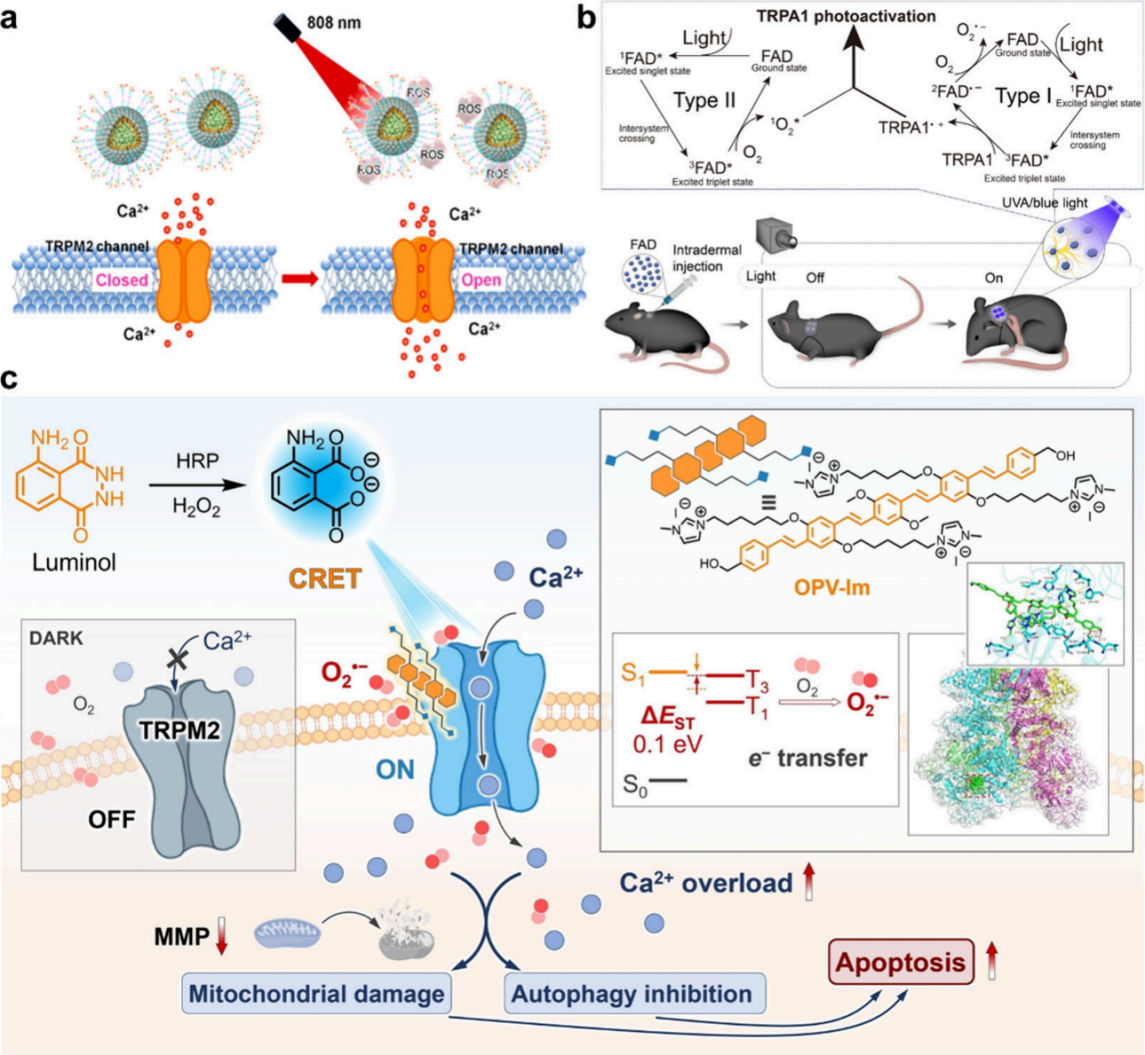
Photodynamic regulation of Ca^2+^ channels with CMs. (a) ETTC-based ROS generation system for activating the TRPM2 channel toward enhanced tumor therapy. Reproduced from ref 43. Copyright 2022, American Chemical Society. (b) FAD-based nanoagonist for remote neurostimulation via photodynamic activation of TRPA1 channels. Reproduced with permission from ref 45. Available under a CC BY-NC license. Copyright 2024, The American Association for the Advancement of Science. (c) External-light-free OPV-Im-based CRET system for *in situ* photoactivation of the TRPM2 channel in living cells. Reproduced with permission from ref 51. Copyright 2025, John Wiley and Sons.

In contrast to CMs, some natural products with an inherent conjugated backbone have also been verified for the activation of ROS-sensitive Ca^2+^ channels. Wang et al. identified flavin adenine dinucleotide (FAD) as photopharmacological agonists through the target-based screening method (Figure 1b).^45^ With the inherent conjugated fused-isoalloxazine rings, excited triplet FAD (^3^FAD*) can either sensitize surrounding O_2_ to generate singlet oxygen (^1^O_2_) or undergo ^3^FAD*-induced free radical reactions with amino acid residues in TRPA1 proteins under blue light irradiation, thus enabling the photoactivation of TRPA1 in sensory neurons. Aiming to increase the tissue penetration depth, a spectra-matchable UCNP–FAD hybrid system was fabricated, developing a NIR-light-activatable nanoagonist. Following the intrathecal injection of UCNP–FAD, the customized noninvasive NIR stimuli remotely activated the spinal TRPA1, achieving the precise bidirectional control of nociception in mice, namely, pain hypersensitivity and/or antinociceptive effects. By synergistically integrating photopharmacology with nanotechnology, the NIR-light-activatable ion channel agent and methodology was provided, which enabled noninvasive neuromodulation, also demonstrating the universal applicability of the photodynamic effect for regulation of Ca^2+^ channels.

Despite these achievements,^43–45^ most of the photodynamic strategies typically require an external light source, severely limiting the practical applications in the real deep tissues and/or organs. Bioluminescent and electro-chemiluminescent energy transfer systems have been pioneered,^25,46–50^ revolutionizing the fields of bioimaging, diagnosis, and therapy in an external-light-free manner. Wang et al. advanced a CM-based chemiluminescence resonance energy transfer (CRET) system for *in situ* photoactivation of TRPM2 in living cells (Figure 1c).^51^ In the presence of horseradish peroxidase (HRP) and H_2_O_2_, luminol spontaneously emitted remarkable blue chemiluminescence, which was further absorbed by an oligo­(*p*-phenylenevinylene) derivative bearing cationic side chains with imidazolium quaternary ammonium terminals (OPV-Im), thus being excited followed by undergoing the promoted ISC process and then sensitizing O_2_ to preferentially generate superoxide anion (O_2_^•–^) through electron transfer pathways. On the other hand, OPV-Im efficiently accumulated on the cell membranes, facilitating the *in situ* activation of TRPM2 channels by the CRET-induced O_2_^•–^ and then the intracellular Ca^2+^ overload. These programmatic reactions eventually resulted in mitochondrial damage and inhibition of autophagy, enabling cancer cell apoptosis and the growth suppression of 3D cancer spheroids. This CM-based CRET system offers an original strategy for activation of Ca^2+^ channels, which gets rid of the external light sources, significantly promoting the controllable precision and partly resolving the issue of light penetration.

The conjugated-molecule-based chemiluminescence resonance energy transfer system offers an original strategy for activation of Ca^2+^ channels, which gets rid of the external light sources, significantly promoting the controllable precision and partly resolving the issue of light penetration.

## PHOTOTHERMAL REGULATION OF the Ca^2+^ CHANNEL

Temperature has been emerging as another promising stimuli that can achieve the regulation of Ca^2+^ channel activities, which typically relies more on the temperature-sensitive Ca^2+^ channels, such as the TRP family, in addition to inducing the membrane capacitance changes. For example, TRPV1-V4 have been revealed to be activated by the heat with temperatures ranging from 27 to 52 °C.^52^ CMs can absorb light and convert to thermal energy through the nonradiative relaxation pathways, and remarkable achievements have been accomplished to obtain CMs with high photothermal conversion efficiency (PCE),^26,27^ allowing for the regulation of Ca^2+^ channels in a photothermal manner. Pu et al. has pioneered the investigations of CM nanoparticles for precise manipulation of living neural activities by targeted photothermal activation of Ca^2+^ channels (Figure 2a).^32^ A NIR-absorbing semiconducting polymer (SP) with narrow-gap diketopyrrolopyrrole units in the backbone was rationally designed and synthesized via Stille polymerization, exhibiting NIR absorption as well as high PCE and stability. Water-dispersible nanoparticles were subsequently fabricated with amphiphilic polymer polystyrene-*b*-poly­(acrylic acid) (PS-PAA), followed by surface conjugation with anti-TRPV1 antibody, thereby enabling precise targeting of thermosensitive TRPV1 channels on neuron membranes. With this photothermal nanomodulator, intracellular Ca^2+^ influx of neuronal cells was activated specifically and rapidly, providing a CM-based precise photothermal strategy without genetic transfection. A similar study was also reported using CMs as photothermal conversion elements for activating the TRPV1 channel to remotely control the transgene system for thrombolysis therapy.^53^ On the other hand, CM nanoparticles can also be applied as nanocarriers for delivery and photothermally triggered release of agonists (i.e., capsaicin for TRPV1)^54^ and/or inhibitor (i.e., curcumin for TRPM2),^55^ presenting a multimodality photothermal–pharmacological approach for regulation of Ca^2+^ channels.

**Figure 2:**
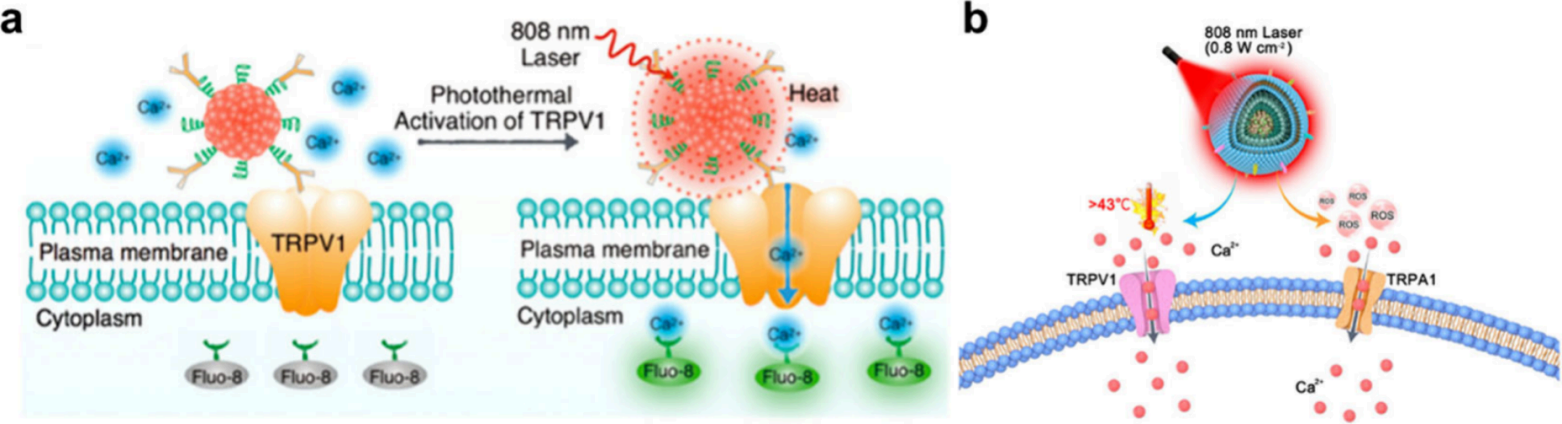
Photothermal regulation of Ca^2+^ channels with CMs. (a) NIR-I light absorbing SP-NPs for photothermal activation of the TRPV1 channel. Reproduced from ref 32. Copyright 2016 American Chemical Society. (b) NIR-I light absorbing S81CF-NPs with the capacity of dual activation of TRPV1 channels for precise regulation of blood glucose homeostasis. Reproduced from ref 56. Copyright 2023 American Chemical Society.

Conjugated molecules can absorb light and convert to thermal energy through the nonradiative relaxation pathways, allowing for the regulation of Ca^2+^ channels in a photothermal manner.

The photothermal manipulation approach can also be integrated with the second optical modulation stimuli, providing a synergistic boosting effect, namely, “two-pronged activation”. Xing et al. developed a dual-activation strategy toward Ca^2+^ channels using NIR-responsive CM nanoparticles for precise regulation of blood glucose homeostasis (Figure 2b).^56^ A NIR-absorbing CM with “A–D” type structure, S81CF, was synthesized through a Knoevenagel condensation reaction. Notably, flexible and rigid moieties were coupled into S81CF chemical structures, which impedes the severe intermolecular stacking, thereby in-part preventing the aggregation-caused fluorescence quenching and facilitating ROS generation. Triggered by an 808 nm laser, S81CF nanoparticles efficiently produced localized heat and ROSs, which simultaneously activated thermosensitive TRPV1 and ROS-sensitive TRPA1, demonstrating dual-channel activation capability. The subsequent Ca^2+^ influx induced endocrine cells to secrete glucagon-like peptide-1 (GLP-1), effectively regulating blood glucose levels. *In vivo* studies demonstrated that such a system successfully maintained glucose homeostasis in diabetic mouse models, exhibiting multiple therapeutic benefits including improved glycemic control, amelioration of obesity-related symptoms, and excellent biosafety. This work provides a remarkable photothermal–photodynamic strategy for diabetes therapy using the Ca^2+^ channel as targets, offering a noninvasive and spatiotemporally precise approach to metabolic disorder management.

The second near-infrared light (NIR-II light, 1000–1700 nm) exhibits reduced tissue scattering and deeper penetration depth, in comparison to the visible and far-red, and even NIR light in the first region (700–1000 nm).^57^ Some optical fibers can be implanted into tissues to provide a deep illumination but might suffer from the invasive systemic damage, especially to the brains, and restrained animal movements.^58^ NIR-II active photothermal neuromodulation with noninvasiveness and deep tissue penetration depth can thus provide an ideal wireless tool to interrogate and manipulate neural activities. Hong and Pu et al. reported macromolecular infrared nanotransducers for deep-brain stimulation (MINDS) in implant- and tether-free animals via wide-field illumination in the NIR-II window.^33^ NIR-II active CM, poly­(benzobisthiadiazole-*alt*-vinylene, pBBTV) with the adjusted “D–A” type structure, was synthesized and participated with poly­(lactide-*co*-glycolide)-*b*-poly­(ethylene glycol) (PLGA-PEG). In addition to the biocompatibility and stability, the fabricated nanotransducers also demonstrated high PCE of 71% at 1064 nm, the wavelength at which exhibits the attenuated scattering in brain tissue, being considered as the most suitable light currently. Upon stereotactical injection and wide-field NIR-II illumination, MINDS certainly activated the ectopically expressed TRPV1 in neurons located at the hippocampus, motor cortex, and ventral tegmental area of mice brains. Notably, the neurons can even be reliably activated with the NIR-II light source positioned over 50 cm above the animal head and with an incident power density of 10 mW mm^–2^. With the achieved modulation of deep-brain neural activities of freely moving mice in a tether-free and implant-free manner, this strategy can potentially allow for behavioral and social interaction studies in a large space area.

## SUPRAMOLECULAR REGULATION OF the Ca^2+^ CHANNEL

Cell membrane potential variation has been considered as an alternative approach for regulating ion channels.^59^ Nanomaterials with the intrinsic surface potential can tune the cell membrane potential and then activate the ion channels without extra stimuli, circumventing the localized oxidative and thermal damage during the photothermal manipulations.^60^ CMs with ionized pending groups thus meet the requirement of such charged nanomaterial.^20,25^ On the other hand, synthetic CMs with defined chemical structures have enabled the supramolecular host–guest complexation with macrocyclic host molecules as well as the elucidation of structure–property relationships, clearly being distinguished with inorganic nanomaterials.^28^ Importantly, CM-based supramolecular systems exhibit remarkable controllability and reversibility,^28^ demonstrating great promise for regulation of Ca^2+^ channels in an external-stimuli-free and switchable manner.

Wang et al. pioneered the concept of “supramolecular genetics”, achieving the supramolecular regulation of cell membrane potential via a switchable system for activating Ca^2+^ channels (Figure 3).^34^ A CM with intrinsic surface potential, namely, OPV with four cationic terminal alkynyl groups, was designed and synthesized to form supramolecular complexes with cucurbit[7]­uril (OPV/4CB[7]), whose hydrophobic cavity and negatively charged potential enabled the efficient host–guest complexation with cationic OPV. The positive charges of OPV were shielded by CB[7] in the supramolecular complexes, displaying an “OFF” effect to the Ca^2+^ channels. However, the assembled architectures were completely dissociated after the addition of competitive molecule adamantylamine hydrochloride (AD) with higher binding constant to CB[7], which released and exposed the terminal cationic alkynyl groups of OPV followed by affecting the cell membrane potential, thus switching “ON” TRPV1 channels. Hierarchically, Cu-catalyzed Click reaction between alkynyl groups on OPV and azide-functionalized cationic oligo-lysines further enhanced positive charge density and resulted in marked membrane potential variations, remarkably amplifying the activation of TRPV1 channels. By introducing Ca^2+^-responsive tumor suppressor genes into cells in advance, the Ca^2+^ influx from TRPV1 activation markedly upregulated p53 expression, eventually leading to cancer cell apoptosis. This work originates the idea of a supramolecular system that enables switchable regulation of Ca^2+^ channels through controllable modulation of intrinsic surface potential without extra stimuli, also offering a Ca^2+^-channel-involved signaling cascade of “membrane potential-Ca^2+^ concentration-gene expression” for cancer therapy.

**Figure 3:**
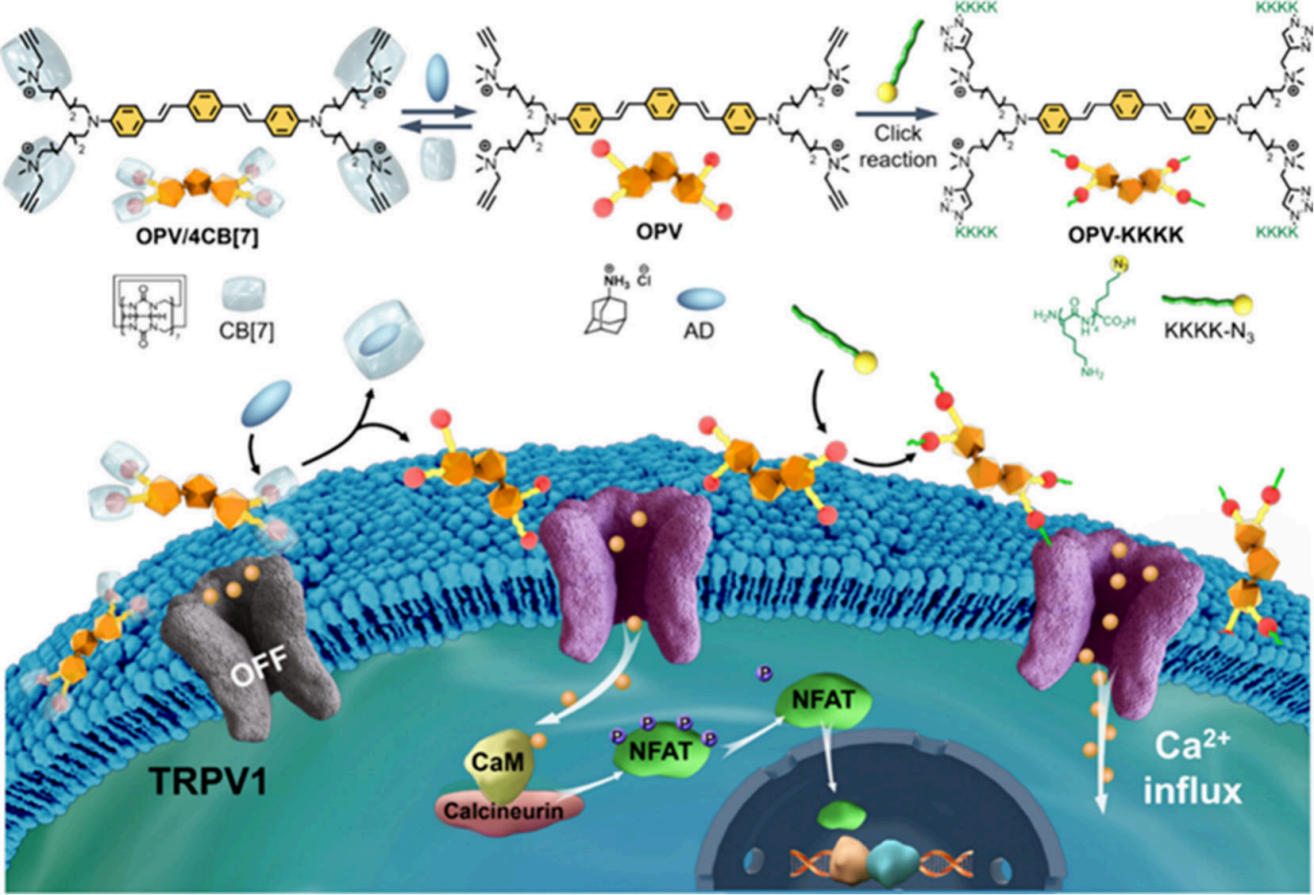
Supramolecular regulation of the TRPV1 Ca^2+^ channel with the OPV/4CB[7] system. Reproduced from ref 34. Copyright 2024 American Chemical Society.

The conjugated-molecule-based supramolecular system can enable switchable regulation of Ca^2+^ channels through controllable modulation of intrinsic surface potential without extra stimuli.

## SUMMARY AND OUTLOOK

CMs have demonstrated tunable optoelectronic properties, intrinsic bioactivities, and flexible assembly characteristics, enabling intrabuilding applications in the regulation of Ca^2+^ channels (Table 1). Their defined chemical structures, excellent biocompatibility, and low systemic toxicity constitute the essential foundation for manipulating Ca^2+^ channels in living cells. On the other hand, CMs exhibited robust external-light-reliable or -free photodynamic and NIR-I/II active photothermal effects, which mediates the oxidative stress- and/or heat-induced activation of Ca^2+^ channels including TRPV1, TRPA1, and TRPM2, thus allowing for the on-demand modulating biological functions, including cancer and thrombolysis therapy, remote neurostimulation, and glycemic management. Notably, some CMs with intrinsic charges unexpectedly activated TRPV1 through inducing cell membrane potential variations, and the reversibility was also achieved with supramolecular chemistry, being proposed as “supramolecular genetics”. This method remarkably advances the adventure of *in situ* switchable regulation of Ca^2+^ channels in an external-stimuli-free manner, contributing to circumvent the critical penetration issue in optogenetics. Overall, the CM-based photodynamic, photothermal, and supramolecular regulation of Ca^2+^ channels has been demonstrated, taking a big step forward to manipulate ion channels for modulating biological functions.

**Table 1:** Summary of Principles, Advantages, and Limitations of Photodynamic, Photothermal, and Supramolecular Manners Using CMs for Regulation of Ca^2+^ Channels

Ca^2+^ Channel Regulation Manner	Principles	Advantages	Limitations
Photodynamic	Photosensitizing CMs generate ROSs to activate ROS-sensitive Ca^2+^ channels	High spatiotemporal accuracy, possibility to be applied in an external-light free manner	Systemic toxicity due to the localized ROS, long response time, limited tissue penetration depth
Photothermal	Photothermal CMs produce heat to activate temperature-sensitive Ca^2+^ channels	High spatiotemporal accuracy, remote wireless manipulation, high tissue penetration depth	Systemic toxicity due to the localized heat, long response time
Supramolecular	Intrinsically charged CMs directly induce the variations of cell membrane potential to activate Ca^2+^ channels	Simple to use without extra stimuli, high biocompatibility, multifunctional controllability	Low spatiotemporal accuracy, lack of cell/tissue targeting capability.

Despite the significant achievements, there are still some critical limitations to be addressed, for CM-based regulation of Ca^2+^ channels toward more practical applications. For example, the response time of current CM-based optical manipulation methods is at second-level (∼s), which is much longer than that of most optogenetic approaches (∼ms), being tricky to study the transient neural dynamics.^33^ On the other hand, the ROSs and/or thermal diffusion potentially increase the risk to cause systemic damage, especially to brain tissues, urgently necessitating shorter response time. In addition to rationally designing and synthesizing CMs with high photosensitization and photothermal conversion capabilities through backbone and side-chain engineering, the integration of sonic, electronic, and mechanical stimulus with optical manipulation using CMs as conversion elements provides an alternative multiplexing method to achieve fast response. Afterward, the CRET-based optogenetics and so-called supramolecular genetics can in part resolve the tissue penetration depth issue by *in situ* stimulation, but still in their infancy. The engineering of such systems with delivery nanocarriers (i.e., liposomes, emulsions, and coacervates)^47,61,62^ to realize *in vivo* barrier penetration, microenvironment-responsive release, tissue/cell/Ca^2+^ channel-targeting, and self-reporting functions, is anticipated to increase the regulation precision, thereby expanding the real application scenarios in the near future. In addition, it should be noted that currently existing CM-based regulations are mainly restrained on TRPV1, TRPA1, and TRPM2 and the manipulation of more Ca^2+^ channels can be further explored, with the subsequent goals to regulate multiple channels simultaneously for boosting biological functions.^63,64^ Considering the biosafety of CMs toward clinical translations, systematic evaluation of their biometabolic pathways, biodegradation processes, and thus clear elucidation of the structure–activity relationships for guiding the high-throughput development of biocompatible CMs are also the key. Therefore, developing new CM systems with fast response time, multimodal responsiveness, targeted precision, and biosafety can further the adventure to efficiently regulate Ca^2+^ as well as other ion channels, thus facilitating modulation of the biological functions and contributing to interdisciplinary research at the interfaces of chemistry, life, and health.
